# Impaired decidual natural killer cell regulation of vascular remodelling in early human pregnancies with high uterine artery resistance

**DOI:** 10.1002/path.4057

**Published:** 2012-07-18

**Authors:** Rupsha Fraser, Guy StJ Whitley, Alan P Johnstone, Amanda J Host, Neil J Sebire, Baskaran Thilaganathan, Judith E Cartwright

**Affiliations:** 1 Division of Biomedical Sciences, St George's, University of LondonLondon, UK; 2 University College London Institute of Child HealthLondon, UK; 3 Fetal Medicine Unit, St George's HospitalLondon, UK

**Keywords:** decidua, natural killer cell, pregnancy, spiral artery, trophoblast, pre-eclampsia, invasion, apoptosis

## Abstract

During human pregnancy, natural killer (NK) cells accumulate in the maternal decidua, but their specific roles remain to be determined. Decidual NK (dNK) cells are present during trophoblast invasion and uterine spiral artery remodelling. These events are crucial for successful placentation and the provision of an adequate blood supply to the developing fetus. Remodelling of spiral arteries is impaired in the dangerous pregnancy complication pre-eclampsia. We studied dNK cells isolated from pregnancies at 9–14 weeks' gestation, screened by uterine artery Doppler ultrasound to determine resistance indices which relate to the extent of spiral artery remodelling. dNK cells were able to promote the invasive behaviour of fetal trophoblast cells, partly through HGF. Cells isolated from pregnancies with higher resistance indices were less able to do this and secreted fewer pro-invasive factors. dNK cells from pregnancies with normal resistance indices could induce apoptotic changes in vascular smooth muscle and endothelial cells *in vitro*, events of importance in vessel remodelling, partly through Fas signalling. dNK cells isolated from high resistance index pregnancies failed to induce vascular apoptosis and secreted fewer pro-apoptotic factors. We have modelled the cellular interactions at the maternal-fetal interface and provide the first demonstration of a functional role for dNK cells in influencing vascular cells. A potential mechanism contributing to impaired vessel remodelling in pregnancies with a higher uterine artery resistance is presented. These findings may be informative in determining the cellular interactions contributing to the pathology of pregnancy disorders where remodelling is impaired, such as pre-eclampsia.

Copyright © 2012 Pathological Society of Great Britain and Ireland. Published by John Wiley & Sons, Ltd.

## Introduction

Following implantation in human pregnancy, cytotrophoblasts arising from the outer layer of the blastocyst differentiate into specialized sub-populations with specific roles in ensuring successful placentation. Placental villi are formed from villous cytotrophoblasts which fuse to form syncytiotrophoblasts. Extravillous trophoblasts (EVTs) invade the maternal decidua as far as the first third of the myometrium. The extent of EVT invasion is critical for implantation and remodelling of the spiral arteries and is tightly regulated in both a temporal and a spatial manner.

The uterine blood supply is built up of a branched structure of arteries which decrease in size as they advance through the myometrium and the endometrium, finally giving rise to spiral arteries. During pregnancy, spiral arteries are remodelled into vessels with a much larger diameter, allowing up to a ten-fold increase in blood flow supplied to the intervillous space, where exchange of gases and nutrients takes place across the syncytiotrophoblast to the fetal vessels of the chorionic villi 1.

The decidual environment consists of a complex network of cell types, including those of the maternal immune system. Infiltration of leukocytes begins prior to implantation and in early pregnancy, the major maternal immune cell component of the decidua, comprising approximately 70%, is decidual natural killer (dNK) cells. Other immune cells include macrophages (20–30%) and T cells (<10%). dNK cells are CD56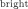, as opposed to peripheral blood natural killer (PB-NK) cells, which are predominately CD56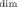. dNK cells are considered a cytokine-producing rather than cytotoxic population, even though they contain the same cytotoxic machinery as PB-NK cells 2.

The roles that dNK cells have in promoting successful placentation in a normal pregnancy are beginning to be elucidated. dNK cells can interact directly with invading EVTs through their expression of inhibitory and activating receptors which bind to trophoblast ligands such as MHC class I molecules 3, as well as producing cytokines, angiogenic factors, and matrix metalloproteinases which can signal to regulate trophoblast invasion 4. In addition to roles in regulating trophoblastic functions, there is increasing evidence to suggest that dNK cells may play an active role in regulating spiral artery remodelling.

Spiral artery remodelling involves the loss of vascular cells and surrounding matrix proteins and occurs in co-ordinated stages. The initiation of vascular remodelling, termed decidua-associated remodelling 1, involves endothelial cell activation and vacuolization, muscular hypertrophy and disorganization, and fibrinoid change 5. These events allow the subsequent trophoblast-dependent remodelling events to occur. Histological studies have suggested an active role for trophoblasts 6, 7, while more recent studies have identified a functional role for EVTs in the remodelling process #b^8^b^9^b^10^b^11^b^12^.

The mechanisms responsible for the earlier decidua-associated vessel changes, which are apparent prior to trophoblast invasion 5, have begun to be studied, although many questions remain unanswered. Immunohistochemical studies have shown that the initial loss of vascular smooth muscle cells (VSMCs) and breaks in the endothelial layer take place in the presence of maternal immune cells but in the absence of invading trophoblasts 13 and that early apoptotic changes can be detected in vascular cells of the spiral artery when leukocytes are present 14. In addition, murine studies have implicated dNK cells in gestational modification of uterine vessels 15, while in the rat, NK cells can influence both the development of uterine spiral arteries and their remodelling 16.

Insufficient remodelling of the spiral arteries with retention of the muscular wall and an inadequate blood supply to the placenta has been associated with the hypertensive pregnancy disorder pre-eclampsia as well as intrauterine growth restriction #b^17^b^18^b^19^. Compelling evidence from several groups identifies dNK cells as key players in controlling the environment at the maternal–fetal interface and there is strong evidence for the involvement of maternal immune cells in the aetiology of pre-eclampsia 20, 21. Human studies are restricted by a lack of access to tissue throughout the key stages of gestation when spiral artery remodelling is taking place. Functional studies using isolated dNK cells have been limited to using first-trimester tissue from terminated pregnancies where the extent of remodelling was not known or using cells isolated from term pre-eclamptic pregnancies or animal models. Hence, reproductive research has been hampered by an inability to study first-trimester cells and tissue from human pregnancies where the extent of spiral artery remodelling is known.

In a normal pregnancy, when vessel remodelling takes place, this leads to increased maternal blood flow through a low-pressure placental bed. However, when remodelling is incomplete, there will be an amplified resistance to maternal blood flow. Doppler ultrasound screening can characterize pregnancies into distinct groups reflecting a proxy measure of the extent to which remodelling of the spiral arteries has occurred 22, which provides a powerful new dimension to studies carried out on early pregnancy tissue. In this study, we have isolated dNK cells from women undergoing surgical termination of pregnancy between 9 and 14 weeks' gestation, who have been classified as having a normal or high uterine artery resistance index, to investigate their role in both the establishment of a healthy pregnancy and the pathogenesis of conditions where remodelling is impaired.

## Materials and methods

### Doppler ultrasound characterization

Maternal uterine artery Doppler velocimetry scans were conducted in the Fetal Medicine Unit, St George's Hospital on women attending clinic for elective termination of pregnancy 22. Ethics Committee approval was in place and all women gave informed written consent. Inclusion criteria were singleton pregnancy, gestational age 9–14 weeks, normal fetal anatomy, and nuchal translucency thickness with no known maternal medical condition or history of recurrent miscarriage. High resistance cases were defined as those with bilateral uterine diastolic notches and a mean resistance index (RI) above the 95th percentile. Normal resistance cases had no diastolic notches and a mean RI below the 95th percentile. These resistance groups represent cases most (21%) and least (<1%) likely to have developed pre-eclampsia had the pregnancy progressed 23, 24. There was no significant difference in gestational ages between the normal-RI and high-RI groups studied.

### Immunohistochemistry

Decidual fragments were formalin-fixed and paraffin-embedded, and 4 µm sections cut. Implantation site fragments were determined by the presence of interstitial EVTs on routine haematoxylin and eosin staining. Decidual tissue was examined from 24 normal-RI and 22 high-RI cases. The median gestational age was 11.5 weeks (range 10.1–13.6 weeks) for normal-RI and 11.1 weeks (range 9.0–12.9 weeks) for high-RI. Immunohistochemical staining was performed for markers of NK cells (CD56; Novacastra, NCL-CD56-504 Novacastra, Newcastle-upon-Tyne, UK) and trophoblast (CK7; Novacastra NCL-L-CK7-560, HLA-G; Serotec MCA2043, Serotec, Kidlington, UK) using standard methods and detection systems with pretreatment where necessary. Full details can be found in the Supplementary methods. Every case was examined at the same magnification and a semi-quantitative estimate of the number/density of NK cells present within the decidua was determined. Immunohistochemical scoring was performed by an experienced pathologist blinded to Doppler information.

### Isolation of dNK cells

Decidual tissue was isolated, washed with HBSS, and dNK cells were isolated using a modification of the methods described previously 25. The purity of cells isolated in this way was examined by flow cytometry. Full details can be found in the Supplementary methods. Purity was 93.6 ± 1.3% (mean ± SEM, *n* = 19 patients). No T cells or macrophages were detected. After 24 and 48 h culture, cells were 80 ± 4% and 77 ± 5% viable (mean ± SEM, *n* = 11 patients), respectively, assessed by Trypan blue exclusion.

### Culture of cell lines

SGHPL-4, a first-trimester human EVT line 26, and SGVSM-9, a human aortic vascular smooth muscle cell line 9, were cultured as previously described. SGHEC-7, a human umbilical vein EC line, was cultured in Medium 199 : RPMI 1640 (1:1) with 10%(v/v) FCS containing 2 mm l-glutamine, 2.5 µg/ml endothelial cell growth supplement (ECGS), 0.09 mg/ml heparin, and 16 mg/ml gentamycin. SGVSM-9 and SGHEC-7 cells have previously been shown to respond in a similar way to vascular cells from dissected spiral arteries 8, 9, 11. SGVSM-9 cells retained differentiated VSMC markers in culture such as α-smooth muscle actin (Supplementary Figure 1). The PB-NK cell line NK92, derived from a non-Hodgkin's lymphoma patient, was cultured in α-MEM containing 5% (v/v) FCS, 5% (v/v) horse serum, 200 U/ml IL-2, 2 mm l-glutamine, 50 IU/ml penicillin, and 50 µg/ml streptomycin.

**Figure 1:**
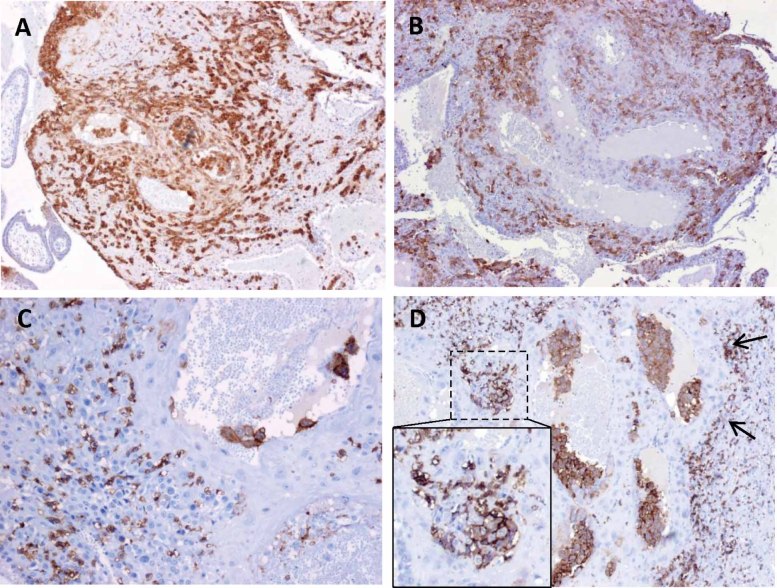
Immunohistochemical analysis of CD56^+^ dNK cell infiltration in decidua basalis. HLA-G staining of EVT (A) and cytokeratin-7 staining of cytotrophoblast (B) confirmed that tissue was obtained from decidua basalis. CD56^+^ dNK cells are shown interstitially and were present in all tissues examined. The extent of CD56 staining was scored. In C and D, CD56^+^ endovascular EVTs can be seen in spiral arteries. Interstitial EVTs are not CD56^+^. (D) CD56^+^ dNK cells can be seen both in close proximity to vessels with endovascular plugs (inset box) and located more distal to plugged vessels (arrows). Original magnification: (A, D) × 20; (B) × 40; (C) × 100

### Time-lapse microscopy

Time-lapse microscopy was carried out using a 1X81 inverted microscope (Olympus, Tokyo, Japan), a digital camera, and motorized stage (Hamamatsu Protonics UK, Welwyn Garden City, UK) within a 37 °C chamber with a 5% CO_2_ atmosphere (Solent Scientific, Segensworth, UK). Images were analysed using ImagePro Plus software (Media Cybernetics, Bethesda, MD, USA). Cell motility and apoptosis were determined using previously described methods 8, 11, 27, with details given in the Supplementary methods.

### Detection of angiogenic and pro-apoptotic factors

A Proteome Profiler Human Angiogenesis Array (R&D Systems, Abingdon, UK) was used according to the manufacturer's instructions on pooled culture supernatants from normal- and high-RI dNK cells (*n* = 28 per group), concentrated 23-fold (VivaSpin columns, 3000 MW cut-off, Sartorius). Densitometric analysis gave relative levels from two array spots/analyte; therefore statistical comparisons could not be made. Levels of cell-associated and soluble FasL, TRAIL, and TNFα were measured in pooled dNK cell lysates or culture supernatants by ELISA (R&D Systems, Peprotech, London, UK). Detection of soluble TRAIL required a 15-fold concentration of supernatants.

### Immunoblotting for cleaved PARP and caspase-3

dNK cells were cultured with VSMCs/ECs in a 1:3 ratio for 30 h. Vascular cells were lysed in RIPA buffer with 1 mm Na_3_VO_4_, 1 mm PMSF, and 60 µg/ml aprotinin. Samples were separated by SDS-PAGE and transferred to a PVDF membrane. After blocking for 1 h, the membrane was incubated with rabbit anti-human cleaved PARP (1/5000; G74A; Promega, Southampton, UK) or anti-cleaved caspase 3 (Cell Signaling Technology, Danvers, MA, USA) or mouse anti-human tubulin (1/10 000; ab7291; Abcam, Cambridge, UK) overnight at 4 °C. Anti-rabbit IgG peroxidase (1/10 000; A6154; Sigma-Aldrich, Dorset, UK) or anti-mouse IgG peroxidase (1/10 000; A4416; Sigma) was added for 1 h at room temperature. Membrane-bound antibodies were detected by enhanced chemiluminescence (ECLPlus; GE Healthcare Life Sciences, Little Chalfont, UK).

### Statistical analysis

One-way ANOVA with Bonferroni's post-test, unpaired *t*-tests or a χ^2^ test was used to determine *p* values using GraphPad Prism. *p* < 0.05 was considered statistically significant.

## Results

### CD56^+^ interstitial dNK cells did not differ between decidua of normal-RI or high-RI pregnancies

Immunohistochemical analysis of decidua basalis [confirmed by the presence of HLA-G (Figure 1A) and cytokeratin-7 (Figure 1B)-positive EVTs] showed that interstitial CD56^+^ dNK cell infiltration was present in all samples examined from both normal and high-RI pregnancies (Figure 1C). In all cases, CD56 immunostaining, when positive, was strong. All cases were examined and an estimate was made of the extent/density of NK cells using a point scale, with slides scored as negative (none or only scattered CD56^+^ cells), positive (representing extensive NK cell infiltration), and plus/minus cases representing those that did not fall at either extreme. No slides scored as negative. Scores were 19 ± and 16 ± for normal versus high-RI, respectively, and 5 + and 6 + scores for normal versus high-RI, respectively. The extent of NK cell infiltration did not differ significantly between decidua from normal-RI or high-RI pregnancies (χ^2^ = 0.26, 1 df, *p* = 0.61, *n* = 24 normal-RI and 22 high-RI samples). In Figures 1C and 1D, CD56^+^ endovascular trophoblasts are shown plugging spiral arteries; up-regulation of CD56 has been characterized in EVTs within spiral arteries 28. CD56^+^ dNK cells could be detected interacting with EVT-containing vessels and distal to vessels (Figure 1D).

### Normal-RI dNK cells induce trophoblast motility to a greater extent than high-RI dNK cells

**Figure 2:**
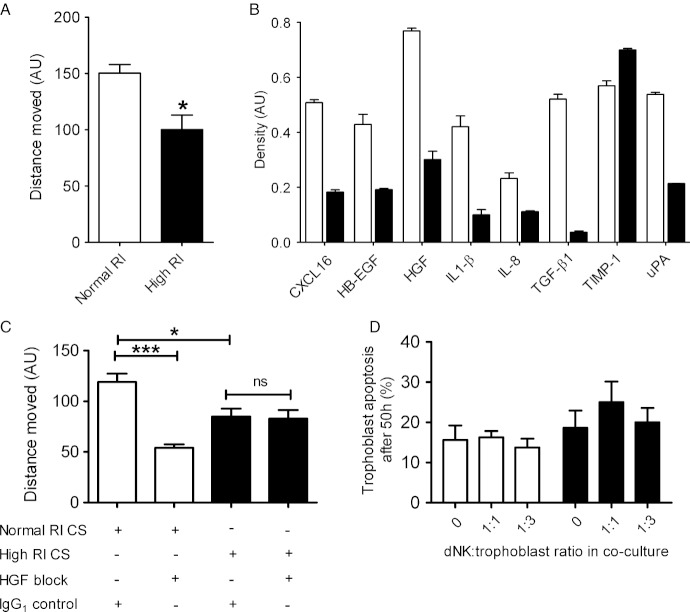
dNK cells induce trophoblast motility but not apoptosis. (A) SGHPL-4 cells were incubated with normal-RI or high-RI dNK cell culture supernatant (pooled from dNK cultures from *n* = 28 patients per group) and cell motility was determined over 24 h. In each experiment, data were expressed relative to the motility induced by culture medium alone [arbitrary units (AU) = 100]. Results are mean ± SEM of pooled data from four separate experiments carried out in duplicate with 40 cells analysed per sequence. *p = 0.016. The median gestational age was 11.3 weeks (range 9.3–13.7 weeks) for normal-RI and 10.9 weeks (range 9.1–13.3 weeks) for high-RI cells used to generate pooled culture supernatants (*p* = 0.4, *t*-test). (B) Normal-RI or high-RI dNK cell culture supernatant (pool of *n* = 28 as above) was concentrated 23-fold and examined by a Proteome Profiler Angiogenesis Array Kit. Mean + range of densitometric analysis of selected spots are shown. Open bars are normal-RI dNK CS; shaded bars are high-RI dNK CS. (C) SGHPL-4 cells were incubated with normal-RI or high-RI dNK cell culture supernatant (as above) + /− 0.3 µg/ml HGF neutralizing antibody or IgG_1_ control, and cell motility was determined over 24 h. Results are mean ± SEM of three separate experiments carried out in duplicate with 40 cells analysed per sequence. *p < 0.05; ***p < 0.0001. (D) SGHPL-4 cells were co-cultured with normal-RI (open bars) or high-RI (shaded bars) dNK cells (from four individual patients) and apoptotic morphology was monitored by time-lapse microscopy for 50 h. Results are mean ± SEM of four separate experiments carried out in duplicate with 40 cells analysed per sequence

Cellular motility is an important component of trophoblast invasion 27. Motility of the EVT cell line, SGHPL-4, was assessed by time-lapse microscopy. SGHPL-4 cells treated with normal-RI dNK culture supernatant showed a 1.5-fold increased motility in comparison with cells treated with high-RI dNK culture supernatant (Figure 2A, *p* = 0.015). The motility with high-RI culture supernatant did not differ from treatment with control medium (not conditioned by culture with dNK cells). An initial screen by proteome array analysis was carried out on pooled culture supernatants from normal- versus high-RI dNK cells to determine factors that may be responsible for regulating motility/invasion to target in function-perturbing experiments. Increased levels of CXCL16, HB-EGF, HGF, IL-1β, IL-8, TGF-β1, and urokinase-type plasminogen activator were detected in culture supernatants from normal-RI dNK, whereas decreased tissue inhibitor of metalloproteinase (TIMP)-1 was detected compared with high-RI dNK culture supernatants (Figure 2B). The levels of all additional proteins assayed are shown in Supplementary Table 1. HGF stimulates EVT motility 27; therefore a HGF blocking antibody was used to examine if HGF was involved in the motility induced by pooled normal-RI dNK culture supernatants. Blocking HGF significantly decreased SGHPL-4 motility compared with cells treated with an isotype control (Figure 2C, *p* < 0.0001), whereas there was no difference in SGHPL-4 motility when treated with high-RI dNK culture supernatant plus Ig control compared with when the HGF blocking antibody was added (Figure 2C).

### dNK cells do not induce trophoblast apoptosis

Increased trophoblast apoptosis has been associated with pregnancies where remodelling is impaired 29. Since dNK cells may have interactions with EVTs which are cytotoxic/apoptotic, the effect of dNK cells on SGHPL-4 cells was monitored by time-lapse microscopy over 50 h. There was no increase in apoptosis above basal levels when in co-culture with normal- or high-RI dNK cells (Figure 2D).

### Normal-RI dNK cells induce vascular cell apoptosis

**Figure 3:**
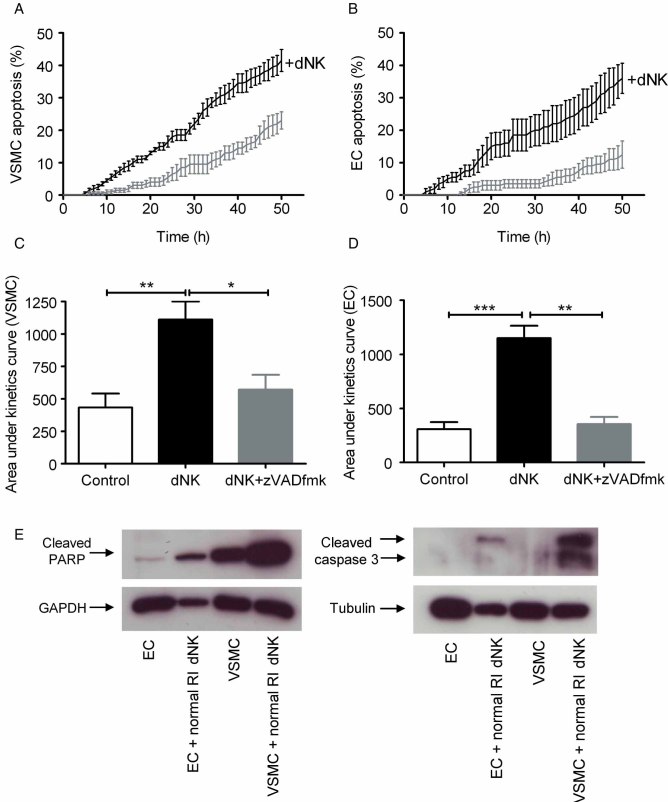
dNK cells induce vascular cell apoptosis. SGVSM-9 or SGHEC-7 cells were co-cultured with normal-RI dNK cells from individual patients in a dNK : vascular cell ratio of 1:3 and monitored over 50 h by time-lapse microscopy. Kinetics of VSMC apoptosis (A) and EC apoptosis (B) induced by normal-RI dNK cells. SGVSM-9 or SGHEC-7 cells were co-cultured alone (control) or with normal-RI dNK cells + /− 50 µm zVAD-fmk over 50 h, and area under the kinetics curve data were generated for VSMC apoptosis (C) and EC apoptosis (D). Results are mean ± SEM of three separate experiments carried out in duplicate with 40 cells analysed per sequence. *p < 0.05; **p < 0.001; ***p < 0.0001. (E) Apoptosis was further confirmed by western blot analysis of cleaved PARP (85 kDa) or cleaved caspase 3 (17/19 kDa) in SGVSM-9 or SGHEC-7 cells cultured with normal-RI dNK cells in a dNK : vascular cell ratio of 1:3 for 30 h. GAPDH (37 kDa) or tubulin (55 kDa) was detected as loading control

The effect of co-cultured normal-RI dNK cells (from individual patients) on VSMCs and ECs was monitored by time-lapse microscopy. VSMC apoptosis was increased by 18.5% after 50 h in the presence of dNK cells (Figure 3A). Incubation of ECs with normal-RI dNK cells led to an increase in apoptosis of 23.5% after 50 h (Figure 3B). Analysis of the area under the kinetics curves showed that incubation of VSMCs with normal-RI dNK cells led to a 2.6-fold increase in apoptosis after 50 h (Figure 3C, *p* < 0.001). The broad spectrum caspase inhibitor zVAD-fmk inhibited the dNK-induced VSMC apoptosis back to almost basal levels (Figure 3C, *p* < 0.05). A similar inhibitory effect was seen when experiments were repeated with SGHEC-7 cells. The area under the kinetics curve data indicated that EC apoptosis increased 3.7-fold when co-cultured with normal-RI dNK cells (Figure 3D, *p* = 0.0001). zVAD-fmk inhibited the dNK-induced EC apoptosis back to basal levels (Figure 3D, *p* < 0.001). Western blot analysis of ECs or VSMCs which had been co-cultured with normal-RI dNK cells showed increased cleavage of PARP and caspase 3 (Figure 3E).

### High-RI dNK cells do not induce vascular cell apoptosis

**Figure 4:**
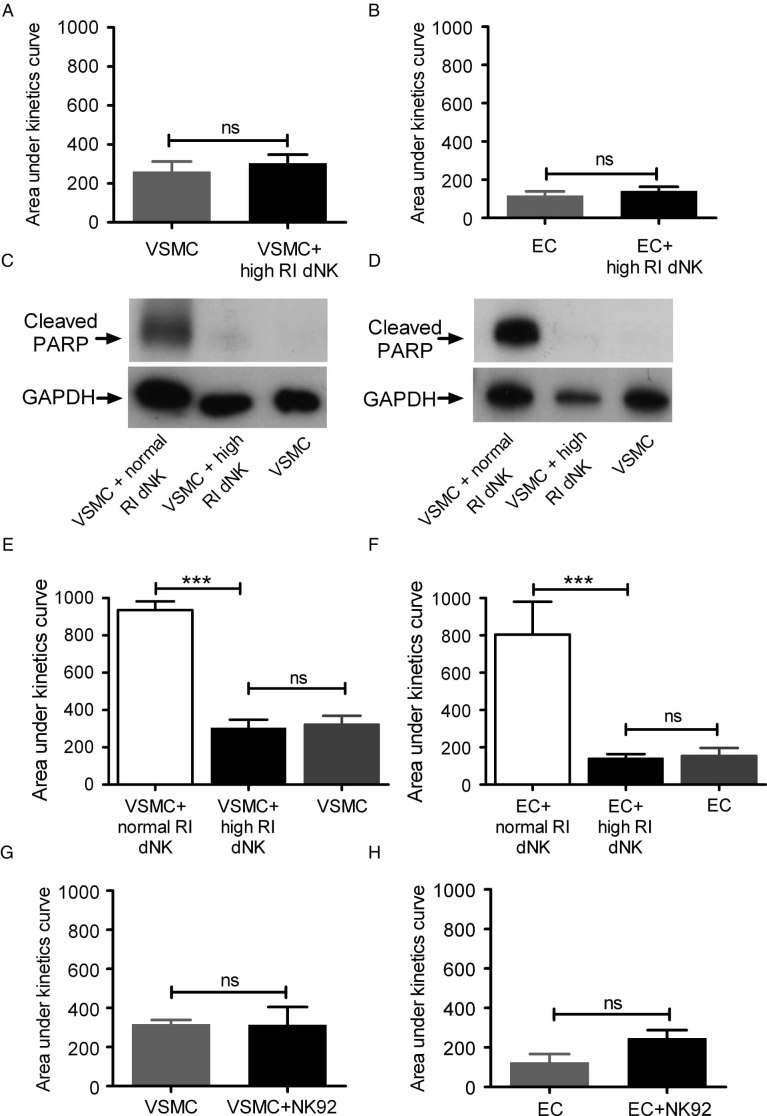
High-RI dNK and NK92 cells do not induce vascular cell apoptosis. SGVSM-9 or SGHEC-7 cells were co-cultured with high-RI dNK cells from individual patients or NK92 cells in a NK : vascular cell ratio of 1:3 and monitored over 50 h by time-lapse microscopy. Results are mean ± SEM of experiments carried out with five separate dNK isolates in duplicate with 40 cells analysed per sequence. VSMC apoptosis (A) or EC apoptosis (B) after 50 h co-culture with high-RI dNK cells. Western blot analysis of cleaved PARP (85 kDa) in SGVSM-9 (C) or SGHEC-7 cells (D) cultured with normal-RI or high-RI dNK cells in a dNK : vascular cell ratio of 1:3 for 30 h. GAPDH (37 kDa) was detected as loading control. VSMC apoptosis (E) and EC apoptosis (F) compared between normal- and high-RI dNK cells. ***p < 0.0001; *n* = 5 separate experiments for each (normal-RI dNK cells were from additional experiments to those shown in Figure 3). The median gestational age of the samples used to generate dNK cells for VSMC co-culture (E) was 12.2 weeks (range 10.1–13.3 weeks) for normal-RI and 10.7 weeks (range 9.3–12.6 weeks) for high-RI (*p* = 0.3, *t*-test). The median gestational age of the samples used to generate dNK cells for EC co-culture (F) was 11.4 weeks (range 9.4–12.7 weeks) for normal-RI and 10.4 weeks (range 9.1–11.7 weeks) for high-RI (*p* = 0.6, *t*-test). VSMC apoptosis (G) or EC apoptosis (H) after 50 h co-culture with NK92 cells; experiments were repeated 3–4 times

The effect of co-culturing high-RI dNK cells (from individual patients) with VSMCs and ECs was monitored by time-lapse microscopy. The area under the kinetics curve data indicated that neither VSMC apoptosis (Figure 4A) nor EC apoptosis (Figure 4B) increased in co-culture with high-RI dNK cells. Western blot analysis of cleaved PARP after co-culture with dNK cells confirmed this result (Figures 4C and 4D). A comparison of the ability of normal-RI dNK and high-RI dNK cells to induce vascular cell apoptosis demonstrated significant differences in apoptosis induction with high versus normal-RI dNK cells on VSMCs (Figure 4E, *p* < 0.001) and ECs (Figure 4F, *p* < 0.001). Neither VSMC apoptosis (Figure 4G) nor EC apoptosis (Figure 4H) increased in co-culture with the PB-NK cell line NK92.

### Normal-RI dNK-induced vascular cell apoptosis is partly a FasL but not TRAIL- or TNF-dependent effect

In order to identify dNK-secreted pro-apoptotic factors, FasL, TNFα, and TRAIL were measured by ELISA in pooled normal-RI or high-RI culture supernatants. Cell-associated factors were assessed in lysates pooled from dNK cultures. Values shown are mean ± range of replicate measurements within ELISAs; therefore statistical comparisons could not be made. Cell-associated FasL was at similar levels in normal-RI dNK (120.1 ± 0.1 pg/mg cell protein, *n* = 28 patients) and high-RI dNK cells (123.8 ± 1.3 pg/mg cell protein, *n* = 28 patients). In contrast, FasL secreted by high-RI dNK cells was reduced in comparison with normal-RI dNK cells (3455 ± 69 and 5766 ± 253 pg secreted/mg cell protein, respectively; *n* = 28 patients per group). TNFα secreted by high-RI dNK cells was also reduced in comparison with normal-RI dNK cells (6754 ± 133 and 7949 ± 136 pg secreted/mg cell protein, respectively; *n* = 9 patients per group), as was cell-associated TNFα (high-RI dNK: 14.18 ± 0.30 pg/mg cell protein, normal-RI dNK: 26.07 ± 1.51 pg/mg cell protein; *n* = 16 patients per group). Cell-associated TRAIL was at similar levels in the high versus normal-RI dNK cells (811.8 ± 3.1 pg/mg cell protein compared with 876.5 ± 2.1 pg/mg cell protein, respectively; *n* = 28 patients per group), whereas TRAIL secretion was only detectable from normal-RI dNK cells (1155 ± 38 pg/mg cell protein, *n* = 28 patients per group), with high-RI dNK cells not producing enough TRAIL to be detected within the assay limitations.

**Figure 5:**
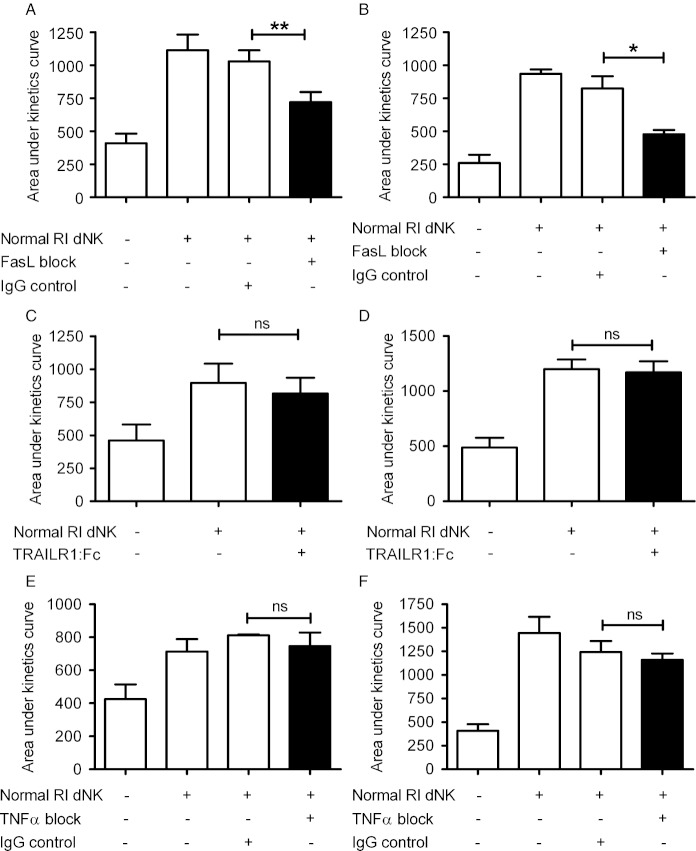
dNK cells induce endothelial cell and vascular smooth muscle cell apoptosis via a FasL-dependent mechanism. SGVSM-9 (A) or SGHEC-7 cells (B) were co-cultured alone (control) or with normal-RI dNK cells + /− 10 µg/ml NOK- 2 (FasL blocking antibody) or control IgG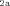 in a dNK : vascular cell ratio of 1:3 and monitored over 50 h by time-lapse microscopy. Results are mean ± SEM of experiments carried out with at least three separate dNK cell isolates in duplicate with 40 cells analysed per sequence. The experiment was repeated with a TRAILR1-Fc construct to block the effects of TRAIL; VSMC (C), EC (D) and a TNFα blocking antibody or control Ig; VSMC (E), EC (F)

To investigate whether FasL, TNFα, or TRAIL was responsible for the normal-RI dNK-induced vascular cell apoptosis, function-perturbing antibodies or constructs were utilized in experiments on cells isolated from individual patients. Addition of a FasL blocking antibody 8 inhibited normal-RI dNK-induced VSMC apoptosis by 50% (Figure 5A, *p* < 0.01) and normal-RI dNK-induced EC apoptosis by 62% (Figure 5B, *p* < 0.05). Addition of a TRAIL-R1Fc construct to inhibit TRAIL signalling 11 (Figures 5C and 5D) or a TNFα blocking antibody (Figures 5E and 5F) had no effect on the induction of apoptosis in either VSMCs or ECs.

## Discussion

There has been considerable interest in the role of immune cells in early pregnancy with studies focusing on the possible role they can have in recurrent miscarriage and early pregnancy loss 30. Investigation of immune cells in early human pregnancy has been hampered by an inability to determine whether the pregnancies being studied are developing normally. Study of term pregnancies has limitations because it is not possible to relate pathology to first-trimester abnormalities. In this study, use of uterine artery Doppler screening has allowed us to classify first-trimester pregnancies into those with a normal resistance index (reflecting more extensive vessel remodelling) compared with those of high resistance index (reflecting impaired artery remodelling). This provides a powerful tool with which to investigate normal pregnancy and gives the opportunity to make direct comparisons with pregnancies where remodelling is impaired.

NK cells are present in the decidua in large numbers at a time when EVT invasion and spiral artery remodelling are taking place, with their numbers diminishing towards the third trimester 31. Our studies showed that there were no major differences in the number of CD56^+^ cells in first-trimester decidua from pregnancies with high-RI or normal-RI, suggesting that the number of dNK cells present is similar. Reduced numbers of dNK cells have been demonstrated in decidua from pre-eclamptic and fetal growth-restricted pregnancies compared with normal pregnancies 31; however, these studies were carried out at term when the number of dNK cells has decreased. Our studies suggest that if dNK cells are contributing to first-trimester pathophysiology, they may be doing so at a functional level rather than as a result of largely differing numbers of cells present, although subtle differences in their co-localization with other cell types, which may relate to their function, remain to be determined.

dNK cells are the most abundant maternal immune cells in the first-trimester decidua and are localized in close proximity to trophoblasts 32, making them prime candidates for influencing their function. Previous studies have suggested both pro-invasive 4, 33, 34 and anti-invasive 35 effects of dNK cells. We assessed the effect of dNK-derived factors on trophoblast migration and demonstrated that cell migration was promoted, an effect consistent with the dNK secretome profile of many factors known to have stimulatory effects on trophoblasts such as uPA 36, CXCL16 37, HB-EGF 38, HGF 27, IL-1β, and IL-8 34. Other secreted factors could down-regulate the invasive process, such as TIMPs and TGFβ, and so the overall balance between pro- and anti-invasive factors will determine the effect on invading cells. In the present study, we extended this further by separating isolated cells into those from pregnancies with high- and normal-RI. Culture supernatants from dNK cells isolated from normal-RI pregnancies were able to promote migration, whereas trophoblast motility in the presence of medium conditioned by high-RI dNK cells was no higher than that with control medium. Furthermore, high-RI dNK cells secreted lower amounts of the invasion-promoting factors listed above than the normal-RI dNK cells. We have previously demonstrated that there is less plugging of first-trimester spiral arteries by endovascular EVTs in pregnancies with high-RI, which supports there being less trophoblast invasion in the high-RI group 22. We suggest that this may be partly attributable to the lack of a pro-invasive environment in the decidua to which dNK cells contribute. HGF has been implicated in the regulation of trophoblast migration 27. We demonstrated that HGF is partially responsible for the pro-migratory effect induced by normal-RI dNK cells but is not involved in migration induced by high-RI dNK cells. Disruption of the gene for mouse HGF leads to embryonic lethality because of a defect in placental development 39, and HGF levels are lower in term placental tissue from pre-eclamptic pregnancies compared with normal pregnancies 40. The role of dNK-derived HGF warrants further investigation since our studies add to the evidence that HGF is important at the fetal–maternal interface.

The changes that occur during spiral artery remodelling involve alterations in extracellular matrix proteins, loss of contractile VSMCs, and the temporary loss of the endothelial layer. It has been suggested that these events occur in stages, with contributions from both maternal immune cells and fetal trophoblast cells 1. The mechanisms of these changes are beginning to be elucidated #b^8^b^9^b^10^b^11^b^12^, and partly involve the induction of vascular apoptosis, a cellular process that does not elicit an inflammatory response and allows the rapid removal of apoptotic cells by phagocytes, consistent with vessel remodelling as part of a physiological rather than pathological process. Apoptotic markers appeared prior to the presence of trophoblast in vessels in a placental–decidual co-culture model 14, suggesting that immune cells, which accumulate near vessels, may be involved. In this study, we investigated whether dNK cells have roles in regulating this process. dNK cells (from normal-RI pregnancies) co-cultured with vascular cells induced caspase-dependent apoptotic changes. A PB-NK cell line did not have this effect. This is the first demonstration of a direct functional interaction between dNK cells and vascular cells in regulating events of importance in vascular remodelling.

We have previously demonstrated that the signalling events in trophoblast-induced vascular cell apoptosis involve members of the TNF death receptor family 8, 9, 11. Spiral artery vascular cells express Fas and TRAIL-R 8, 11, which, on binding to their ligands, FasL and TRAIL, respectively, lead to apoptosis. dNK cells expressed and secreted FasL, TNFα, and TRAIL. We further demonstrated that Fas–FasL interactions, but not TNFα or TRAIL-mediated events, were partly responsible for dNK-induced apoptosis. Our previous studies showed that Fas–FasL was also important in trophoblast induction of apoptosis 8, 9, highlighting the importance of this signalling pathway in more than one interaction at the maternal–fetal interface. In addition, other cytokines and pro- and anti-angiogenic factors which are known to be produced by dNK cells have recognized roles in regulating vessel integrity 41. In a normal healthy artery, VSMCs are mostly of a functional, contractile phenotype, whereas when they dedifferentiate, a more synthetic, proliferative phenotype is adopted. Dedifferentiation may result in VSMCs which are more prone to migration or apoptosis induction. Recent studies suggest that NK cells can influence the alignment and organization of VSMCs, which may represent an additional effect on their differentiation status 42.

The present study, in addition to what is known about trophoblast-dependent effects, suggests that regulation of uterine vessel remodelling involves a complex interplay between maternal and fetal cell types. We suggest that preliminary stages are initiated by dNK-cell induction of vascular cell changes, including apoptosis, and that this is later completed by trophoblasts. There is likely to be overlap and redundancy in the system as the end result of remodelling is so fundamental for a successful pregnancy. It is also interesting to note that some of the same signalling pathways have now been implicated in regulating remodelling induced by both maternal and fetal cells. NK cells are not as abundant in the inner myometrium as they are in the decidua 1; therefore the relative contribution of maternal and fetal cells will be likely to change depending where along the vessel length remodelling is occurring.

Pre-eclamptic women have spiral arteries which retain more non-pregnant histological features than those with uncomplicated pregnancies 43, 44. Having demonstrated that dNK cells from normal-RI pregnancies have a functional interaction with vascular cells, we extended our investigations to dNK cells from pregnancies with high-RI. We showed that these cells failed to induce vascular apoptosis. Analysis of pro-apoptotic factors revealed that FasL, TRAIL, and TNFα were at lower levels from high-RI than from normal-RI dNK cells, although this remains to be profiled from individual patients. We hypothesize that lower levels of pro-apoptotic factors, in particular FasL, as we associated this with dNK-induced vascular cell apoptosis, may partially explain the decreased effect of dNK cells from high-RI pregnancies.

Cross-talk between dNK cells and both trophoblast and vascular cells is important in regulating development at the maternal–fetal interface. We have demonstrated that maternal immune cells can regulate vascular cell apoptosis, a part of the remodelling process previously shown to involve fetal trophoblasts, and importantly have demonstrated that dNK cells isolated from pregnancies with high uterine artery resistance indices are unable to activate some of these processes. A high resistance uterine artery blood flow is likely to reflect poor placentation and impaired artery remodelling in all of the high-RI group; however, there are clearly maternal and/or fetal adaptations that compensate for this which, in many cases, prevent the development of pregnancy complications such as pre-eclampsia. This highlights the importance of integrating investigations of both the maternal and the fetal components in early pregnancy such that appropriate interventions can be developed to optimize pregnancy success. Our introduction of tools to associate a proxy measure of remodelling with the study of first-trimester cells now means that these investigations will be possible.

## SUPPORTING INFORMATION ON THE INTERNET

The following supporting information may be found in the online version of this article.

**Supplementary methods**

**Figure S1.** α-Smooth muscle actin expression in spiral artery VSMC and SGVSM-9 cells.

**Table S1.** Angiogenic factors secreted by dNK cells.

## References

[1] Pijnenborg R, Vercruysse L, Hanssens M (2006). The uterine spiral arteries in human pregnancy: facts and controversies. Placenta.

[2] Tabiasco J, Rabot M, Aguerre-Girr M (2006). Human decidual NK cells: unique phenotype and functional properties—a review. Placenta.

[3] Moffett-King A (2002). Natural killer cells and pregnancy. Nature Rev Immunol.

[4] Hanna J, Goldman-Wohl D, Hamani Y (2006). Decidual NK cells regulate key developmental processes at the human fetal–maternal interface. Nature Med.

[5] Craven CM, Morgan T, Ward K (1998). Decidual spiral artery remodelling begins before cellular interaction with cytotrophoblasts. Placenta.

[6] Pijnenborg R, Bland JM, Robertson WB (1983). Uteroplacental arterial changes related to interstitial trophoblast migration in early human pregnancy. Placenta.

[7] Kam EP, Gardner L, Loke YW (1999). The role of trophoblast in the physiological change in decidual spiral arteries. Hum Reprod.

[8] Ashton SV, Whitley GS, Dash PR (2005). Uterine spiral artery remodeling involves endothelial apoptosis induced by extravillous trophoblasts through Fas/FasL interactions. Arterioscler Thromb Vasc Biol.

[9] Harris LK, Keogh RJ, Wareing M (2006). Invasive trophoblasts stimulate vascular smooth muscle cell apoptosis by a fas ligand-dependent mechanism. Am J Pathol.

[10] Red-Horse K, Rivera J, Schanz A (2006). Cytotrophoblast induction of arterial apoptosis and lymphangiogenesis in an *in vivo* model of human placentation. J Clin Invest.

[11] Keogh RJ, Harris LK, Freeman A (2007). Fetal-derived trophoblast use the apoptotic cytokine tumor necrosis factor-alpha-related apoptosis-inducing ligand to induce smooth muscle cell death. Circ Res.

[12] James JL, Whitley GS, Cartwright JE (2011). Shear stress and spiral artery remodelling: the effects of low shear stress on trophoblast-induced endothelial cell apoptosis. Cardiovasc Res.

[13] Smith SD, Dunk CE, Aplin JD (2009). Evidence for immune cell involvement in decidual spiral arteriole remodeling in early human pregnancy. Am J Pathol.

[14] Hazan AD, Smith SD, Jones RL (2010). Vascular–leukocyte interactions. Mechanisms of human decidual spiral artery remodeling *in vitro*. Am J Pathol.

[15] Croy BA, He H, Esadeg S (2003). Uterine natural killer cells: insights into their cellular and molecular biology from mouse modelling. Reproduction.

[16] Chakraborty D, Rumi MA, Konno T (2011). Natural killer cells direct hemochorial placentation by regulating hypoxia-inducible factor dependent trophoblast lineage decisions. Proc Natl Acad Sci U S A.

[17] Meekins JW, Pijnenborg R, Hanssens M (1994). A study of placental bed spiral arteries and trophoblast invasion in normal and severe pre-eclamptic pregnancies. Br J Obstet Gynaecol.

[18] Pijnenborg R, Anthony J, Davey DA (1991). Placental bed spiral arteries in the hypertensive disorders of pregnancy. Br J Obstet Gynaecol.

[19] Brosens I, Dixon HG, Robertson WB (1977). Fetal growth retardation and the arteries of the placental bed. Br J Obstet Gynaecol.

[20] Hiby SE, Apps R, Sharkey AM (2010). Maternal activating KIRs protect against human reproductive failure mediated by fetal HLA-C2. J Clin Invest.

[21] Hiby SE, Walker JJ, O'Shaughnessy KM (2004). Combinations of maternal KIR and fetal HLA-C genes influence the risk of preeclampsia and reproductive success. J Exp Med.

[22] Prefumo F, Sebire NJ, Thilaganathan B (2004). Decreased endovascular trophoblast invasion in first trimester pregnancies with high-resistance uterine artery Doppler indices. Hum Reprod.

[23] Melchiorre K, Wormald B, Leslie K (2008). First-trimester uterine artery Doppler indices in term and preterm pre-eclampsia. Ultrasound Obstet Gynecol.

[24] Poon LC, Staboulidou I, Maiz N (2009). Hypertensive disorders in pregnancy: screening by uterine artery Doppler at 11–13 weeks. Ultrasound Obstet Gynecol.

[25] Trundley A, Gardner L, Northfield J (2006). Methods for isolation of cells from the human fetal–maternal interface. Methods Mol Med.

[26] Choy MY, Whitley G, Manyonda IT (2000). Efficient, rapid and reliable establishment of human trophoblast cell lines using poly-l-ornithine. Early Pregnancy: Biol Med.

[27] Cartwright JE, Holden DP, Whitley GS (1999). Hepatocyte growth factor regulates human trophoblast motility and invasion: a role for nitric oxide. Br J Pharmacol.

[28] Burrows TD, King A, Loke YW (1994). Expression of adhesion molecules by endovascular trophoblast and decidual endothelial cells: implications for vascular invasion during implantation. Placenta.

[29] Genbacev O, DiFederico E, McMaster M (1999). Invasive cytotrophoblast apoptosis in pre-eclampsia. Hum Reprod.

[30] Quenby S, Farquharson R (2006). Uterine natural killer cells, implantation failure and recurrent miscarriage. Reprod Biomed Online.

[31] Williams PJ, Bulmer JN, Searle RF (2009). Altered decidual leucocyte populations in the placental bed in pre-eclampsia and foetal growth restriction: a comparison with late normal pregnancy. Reproduction.

[32] Trundley A, Moffett A (2004). Human uterine leukocytes and pregnancy. Tissue Antigens.

[33] Lash GE, Otun HA, Innes BA (2010). Regulation of extravillous trophoblast invasion by uterine natural killer cells is dependent on gestational age. Hum Reprod.

[34] De Oliveira LG, Lash GE, Murray-Dunning C (2010). Role of interleukin 8 in uterine natural killer cell regulation of extravillous trophoblast cell invasion. Placenta.

[35] Hu Y, Dutz JP, MacCalman CD (2006). Decidual NK cells alter *in vitro* first trimester extravillous cytotrophoblast migration: a role for IFN-gamma. J Immunol.

[36] Liu J, Chakraborty C, Graham CH (2003). Noncatalytic domain of uPA stimulates human extravillous trophoblast migration by using phospholipase C, phosphatidylinositol 3-kinase and mitogen-activated protein kinase. Exp Cell Res.

[37] Huang Y, Zhu XY, Du MR (2006). Chemokine CXCL16, a scavenger receptor, induces proliferation and invasion of first-trimester human trophoblast cells in an autocrine manner. Hum Reprod.

[38] Leach RE, Kilburn B, Wang J (2004). Heparin-binding EGF-like growth factor regulates human extravillous cytotrophoblast development during conversion to the invasive phenotype. Dev Biol.

[39] Uehara Y, Minowa O, Mori C (1995). Placental defect and embryonic lethality in mice lacking hepatocyte growth factor/scatter factor. Nature.

[40] Furugori K, Kurauchi O, Itakura A (1997). Levels of hepatocyte growth factor and its messenger ribonucleic acid in uncomplicated pregnancies and those complicated by preeclampsia. J Clin Endocrinol Metab.

[41] Zhou Y, Bellingard V, Feng KT (2003). Human cytotrophoblasts promote endothelial survival and vascular remodeling through secretion of Ang2, PlGF, and VEGF-C. Dev Biol.

[42] Harris LK (2011). Transformation of the spiral arteries in human pregnancy: key events in the remodelling timeline. Placenta.

[43] Brosens IA, Robertson WB, Dixon HG (1972). The role of the spiral arteries in the pathogenesis of preeclampsia. Obstet Gynecol Annu.

[44] Khong TY, De Wolf F, Robertson WB (1986). Inadequate maternal vascular response to placentation in pregnancies complicated by pre-eclampsia and by small-for-gestational age infants. Br J Obstet Gynaecol.

[45] Fickling SA, Tooze JA, Whitley GS (1992). Characterization of human umbilical vein endothelial-cell lines produced by transfection with the early region of Sv40. Exp Cell Res.

[46] Cartwright JE, Whitley GS, Johnstone AP (1995). The expression and release of adhesion molecules by human endothelial cell lines and their consequent binding of lymphocytes. Exp Cell Res.

