# Supplementary material for: Impaired decidual natural killer cell regulation of vascular remodelling in early human pregnancies with high uterine artery resistance
Source: J Pathol. 2012 Jul 18;228(3):322–32. doi: 10.1002/path.4057 (PMC3499663; doi:10.1002/path.4057)
Supplement: Supplementary file 1 [file path0228-0322-SD1.pdf]

Supplementary Figure 1

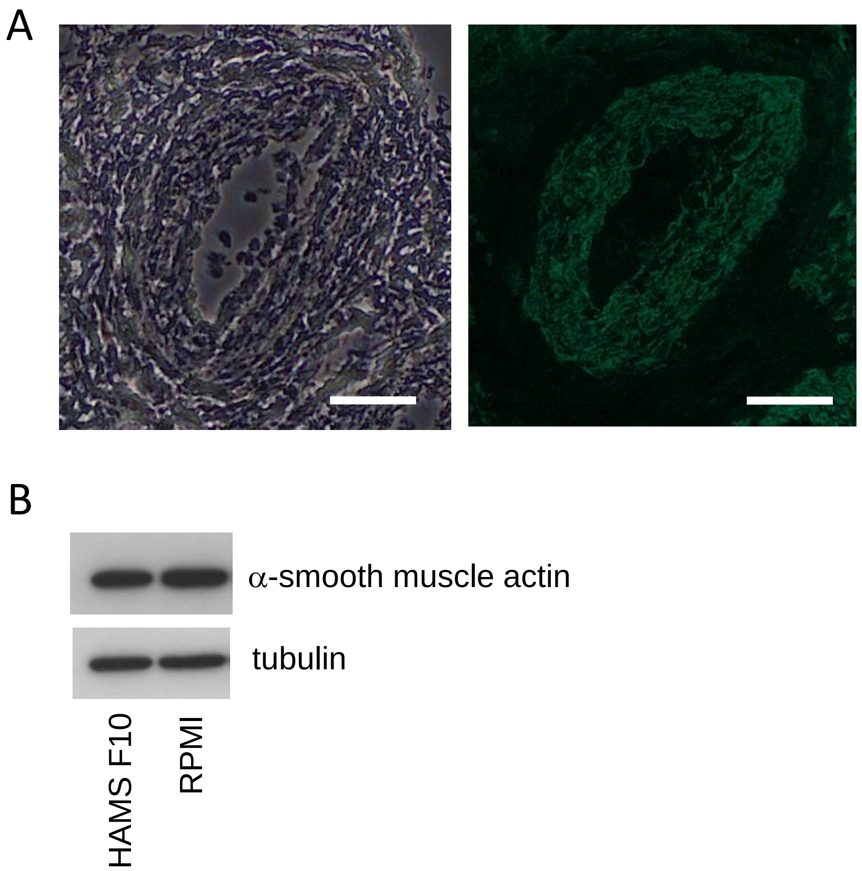

Supplementary Figure 1: α-smooth muscle actin expression in spiral artery VSMC and SGVSM-9 cells.

A: Non-placental bed uterine biopsies were obtained from women undergoing uncomplicated Caesarean sections. Ethical committee approval was in place and written consent was obtained. Spiral arteries were dissected, frozen in embedding compound and cryosectioned. Slides were fixed in 4% paraformaldehyde for 20min, permeabilised using 0.2% Triton X-100 for 5min and blocked in 10% normal goat serum. Sections were incubated in mouse anti-smooth muscle actin (DAKO, 0.1μg/ml) for 1h followed by biotinylated goat anti-mouse Ig (Vector labs, 5μg/ml) for 30min then streptavidin-fluorescein (10μg/ml) for 45min. Phase contrast image of spiral artery (left hand image), α-smooth muscle actin expression (right hand image). Scale bar=50μm.

B: Expression of α-smooth muscle actin (42 kDa) in SGVSM-9 cells cultured in HAMS F10 with 10% FCS or RPMI with 10% FCS was confirmed by western blot analysis. Tubulin (55 kDa) is shown as a loading control.
