# Supplementary figures and images for: Impaired decidual natural killer cell regulation of vascular remodelling in early human pregnancies with high uterine artery resistance
Source: J Pathol. 2012 Jul 18;228(3):322–32. doi: 10.1002/path.4057 (PMC3499663; doi:10.1002/path.4057)

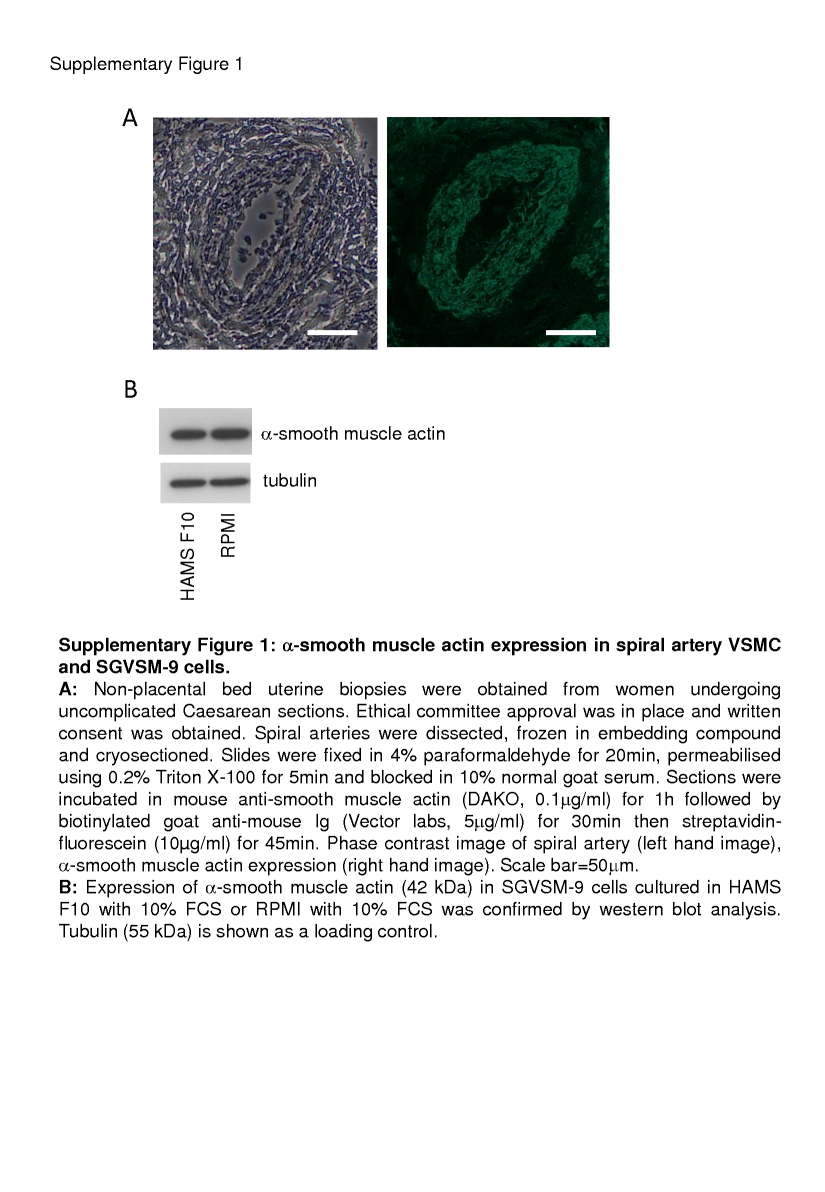

Supplement: Supplementary file 2 [file path0228-0322-SD2.png]
