# Supplementary material for: Impaired decidual natural killer cell regulation of vascular remodelling in early human pregnancies with high uterine artery resistance
Source: J Pathol. 2012 Jul 18;228(3):322–32. doi: 10.1002/path.4057 (PMC3499663; doi:10.1002/path.4057)
Supplement: Supplementary file 4 [file path0228-0322-SD4.doc]

**Supplementary Table 1: Angiogenic factors secreted by dNK cells.**

Normal-RI or high-RI dNK cell culture supernatant (pool of n=28) were concentrated 23-fold and examined by a Proteome Profiler Angiogenesis Array Kit (R&D Systems). Mean±range of densitometric analysis is shown. ND=not detectable (arbitrary density units <0.04 were scored as not detectable).

|  | **Normal-RI dNK CS** | | **High-RI dNK CS** | |
| --- | --- | --- | --- | --- |
| **Protein** | **Mean density (AU)** | **± range** | **Mean density (AU)** | **± range** |
| Activin A | 0.13 | 0.00 | 0.13 | 0.00 |
| ADAMTS-1 | 0.22 | 0.00 | 0.12 | 0.01 |
| Angiogenin | 0.77 | 0.00 | 0.24 | 0.00 |
| Angiopoietin-1 | 0.14 | 0.04 | 0.08 | 0.01 |
| Angiopoietin-2 | 0.12 | 0.00 | 0.07 | 0.00 |
| Angiostatin | 0.10 | 0.01 | 0.06 | 0.00 |
| Amphiregulin | 0.58 | 0.01 | 0.05 | 0.00 |
| Artemin | 0.05 | 0.02 | 0.05 | 0.00 |
| Coagulation Factor III | 0.32 | 0.04 | 0.18 | 0.02 |
| CXCL16 | 0.51 | 0.01 | 0.18 | 0.01 |
| DPPIV | 0.68 | 0.00 | 0.61 | 0.02 |
| EGF | 0.22 | 0.03 | 0.15 | 0.02 |
| EG-VEGF | 0.48 | 0.01 | 0.39 | 0.01 |
| Endoglin | 0.48 | 0.03 | 0.17 | 0.01 |
| Endostatin | 0.38 | 0.03 | 0.11 | 0.00 |
| Endothelin-1 | 0.12 | 0.01 | 0.07 | 0.01 |
| FGF acidic | 0.09 | 0.01 | 0.07 | 0.00 |
| FGF basic | 0.30 | 0.01 | 0.09 | 0.00 |
| FGF-4 | 0.21 | 0.01 | 0.06 | 0.01 |
| FGF-7 | 0.21 | 0.03 | 0.14 | 0.00 |
| GNDF | 0.27 | 0.05 | 0.18 | 0.03 |
| GM-CSF | 0.87 | 0.04 | 0.86 | 0.01 |
| HB-EGF | 0.43 | 0.04 | 0.19 | 0.00 |
| HGF | 0.77 | 0.01 | 0.30 | 0.03 |
| IGFBP-1 | 0.67 | 0.02 | 0.53 | 0.03 |
| IGFBP-2 | 0.73 | 0.02 | 0.55 | 0.03 |
| IGFBP-3 | 0.62 | 0.00 | 0.32 | 0.01 |
| IL-1β | 0.42 | 0.04 | 0.10 | 0.02 |
| IL-8 | 0.23 | 0.02 | 0.11 | 0.00 |
| TGF-β1 | 0.52 | 0.02 | 0.04 | 0.01 |
| Leptin | 1.31 | 0.01 | ND | ND |
| MCP-1 | 0.59 | 0.06 | 0.46 | 0.00 |
| MIP-1α | 0.65 | 0.03 | 0.61 | 0.03 |
| MMP-8 | 0.54 | 0.04 | 0.50 | 0.03 |
| MMP-9 | 0.73 | 0.00 | 0.72 | 0.01 |
| NRG1-β1 | 0.25 | 0.05 | 0.23 | 0.04 |
| Pentraxin 3 | 0.43 | 0.00 | 0.23 | 0.00 |
| PD-ECGF | 0.17 | 0.02 | 0.10 | 0.02 |
| PDGF-AA | 0.20 | 0.00 | 0.04 | 0.00 |
| PDGF-AB/PDGF-BB | 0.13 | 0.01 | 0.04 | 0.00 |
| Persephin | 0.06 | 0.01 | ND | ND |
| Platelet factor-4 | 0.40 | 0.00 | 0.13 | 0.00 |
| PlGF | 0.44 | 0.00 | ND | ND |
| Prolactin | 0.38 | 0.02 | 0.25 | 0.00 |
| Serpin B5 | 0.17 | 0.02 | 0.15 | 0.02 |
| Serpin E1 | 0.34 | 0.04 | 0.39 | 0.01 |
| Serpin F1 | 0.19 | 0.02 | 0.16 | 0.03 |
| TIMP-1 | 0.57 | 0.02 | 0.70 | 0.01 |
| TIMP-4 | 0.10 | 0.00 | ND | ND |
| Thrombospondin-1 | 0.08 | 0.00 | ND | ND |
| Thrombospondin-2 | 0.09 | 0.02 | ND | ND |
| uPA | 0.54 | 0.01 | 0.21 | 0.00 |
| Vasohibin | 0.05 | 0.00 | ND | ND |
| VEGF | 0.30 | 0.01 | 0.37 | 0.01 |
| VEGF-C | ND | ND | ND | ND |
