# Supplementary material for: Impaired decidual natural killer cell regulation of vascular remodelling in early human pregnancies with high uterine artery resistance
Source: J Pathol. 2012 Jul 18;228(3):322–32. doi: 10.1002/path.4057 (PMC3499663; doi:10.1002/path.4057)
Supplement: Supplementary file 5 [file path0228-0322-SD5.doc]

**Supporting information**

**Supplementary methods**

***Immunohistochemistry***

Following fixation and sectioning as described, sections were floated onto 3-aminopropyltriethoxysilane-coated slides and dried overnight at 37°C. The sections were dewaxed and rehydrated. Antigen retrieval was performed as necessary by predetermined experiment for each primary antibody. This entailed either digestion in 0.02% protease in phosphate buffered saline solution at 37°C, pressure cooking in a microwave in citrate/ethylenediaminetetraacetic acid buffer at pH 6.2 for 4 minutes at full pressure, or no pretreatment. The sections were incubated in solutions of the primary antibody for 1 hour at room temperature. An Avidin/Biotin-free detection system was used (DAKO Cytomation EnVision+ system) with 30-minute incubation. Antigen binding was visualized using 5% DAB in dimethyl formamide for 5-10 minutes at room temperature in a fume cupboard. Meyer hematoxylin was used as a counterstain and the sections were then dehydrated, cleared, and mounted in DPX medium and examined with a conventional light microscope.

***Isolation of decidual NK cells***

The decidua was minced finely and incubated in 20ml serum-free HAMS-F10 medium (Invitrogen) containing 0.1mg/ml of DNAse I (20 KU/mg), 2mg/ml of collagenase (type II, 25 KU/ml) and 2.5 μg/ml amphotericin B for 16h at room temperature (RT) with constant turning. The cell suspension was removed from the undigested tissue and filtered sequentially through a 70μm and 100μm filter before centrifugation at 500*g* for 12 minutes at 20°C. The pellet was resuspended in 10ml of red blood cell lysis buffer (8.3 g/L ammonium chloride in 0.01 M Tris-HCl pH7.5) and incubated at RT for 10 minutes and then centrifuged at 500*g* for 12 minutes at 20°C. The cells were washed once with serum-free HAMS-F10 medium and resuspended in 100μl MACS buffer [phosphate buffered saline (PBS; Invitrogen) with 0.5% (w/v) bovine serum albumin (BSA), 2mM EDTA adjusted to pH7.2] per 10^7^ cells and 20μl CD56 antibody coated magnetic beads (Miltenyi Biotec) was added per 10^7^ cells and incubated at 4°C for 20 minutes. Following the incubation, 20ml PBS buffer was added and cells washed by centrifugation at 500*g* for 10 minutes at 20°C. The cells were resuspended in 2ml MACS buffer and separated using an LS column and MidiMACS separator (Miltenyi Biotec) as per manufacturer’s instructions. The CD56^+^ cells were centrifuged at 400*g* for 10 minutes at 22°C and cultured at approximately 6x10^5^ cells/ml in RPMI 1640 medium (Invitrogen) with 10% fetal calf serum (FCS), containing 2.5 μg/ml amphotericin B, 2mM L-glutamine, 50μg/ml penicillin and 50μg/ml streptomycin, 50ng/ml stem cell factor (SCF) and 5ng/ml interleukin (IL)-15 (Peprotech) at 37°C in a 5% CO_2_ humidified incubator. The cells were either used immediately for co-culture studies or cultured for 24h for collection of conditioned medium. After 24h cells were pelleted and lysed for 15 minutes on ice, in PBS containing 1% (v/v) Nonidet P-40, 0.5% (w/v) deoxycholate, 0.1% (w/v) sodium dodecyl sulphate, 1nM Na_3_VO_4_, 1nM phenylmethyl sulfonyl fluoride, and 10 μg/ml aprotinin. Protein concentration was determined by Bradford assay (BioRad) according to manufacturer’s instructions. Conditioned medium was centrifuged for 10 minutes at 700*g* at 4°C to remove debris. Lysates and culture supernatants were stored at -80°C. For some experiments culture supernatants or cell lysates were pooled from 28 normal-RI dNK cultures or 28 high-RI cultures. The median gestational age was 11.3 weeks (range 9.3-13.7) for normal-RI and 10.9 weeks (range 9.1-13.3) for high-RI.

NK cell purity was determined immediately after isolation by labelling with PE-conjugated anti-CD56 (NCAM.16.2, Beckton Dickinson) for NK cells, FITC-conjugated anti-CD14 (Beckman Coulter) for macrophages, and PerCP-conjugated anti-CD3 (eBioscience, SK7) for T cells and flow cytometric analysis (Beckman Coulter). Non-immune IgG_2b_ conjugated to PE (Santa Cruz Biotechnologies) was used as a negative control for anti-CD56, non-immune IgG_2a_ conjugated to FITC (Beckman Coulter) was used as a negative control for anti-CD14, and non-immune IgG_1_ conjugated to PerCP (BD Biosciences) was used as a negative control for anti-CD3. The percentage of cells that were positive (i.e., with a fluorescence intensity above the negative control) was calculated.

***Time-Lapse Microscopy***

*Motility:* SGHPL-4 cells (2x10^4^ cells/ml, 1ml per well, 12 well-plates), were cultured for 6h in Hams-F10 containing 10% (v/v) FCS, then medium was replaced with Hams-F10 containing 0.5% (v/v) FCS for 16h (to induce quiescence by serum starvation). The media was replaced with pooled normal- or high-RI dNK cell culture supernatants. To block HGF activity, 0.3μg/ml HGF neutralising antibody or control IgG_1_ (R&D Systems) was added. Images were captured at 15 minute intervals over 24h. For each sequence, 40 cells were chosen at random and the total distance moved by each cell over 24h was measured.

*Apoptosis:* Time-lapse microscopy to monitor apoptotic morphology is a sensitive method for detecting subtle changes in apoptotic kinetics [8,9,11]. In each experiment, images were captured every 15 minutes over 50h. SGVSM-9 or SGHEC-7cells (3x10^4^ cells/ml and 2.5x10^4^ cells/ml respectively, 1ml per well, 12 well-plates), were cultured for 24h. SGVSM-9 cells were used between passage number 57-73 as described in previous studies [9,11]. SGHEC-7 cells were used between passage number 13 and 23 when they retain a normal EC phenotype as previously described [45,46]. dNK cells or NK92 cells were added at a ratio of 1:3 (dNK/NK92:vascular cell) or no NK additions to the control wells. To block FasL, TRAIL, or TNF-α, 10μg/ml FasL blocking antibody (NOK-2) or control IgG_2a_ (BD Pharmingen), 10μg/ml rhTRAILR1:Fc (Alexis Biochemicals), 15μg/ml TNFα blocking antibody (MAb1, BD Biosciences) or control IgG_1_ (BD Biosciences) were added with normal-RI dNK cells. To confirm caspase-dependent apoptosis, 50μM zVAD-fmk, a broad spectrum caspase inhibitor (Calbiochem), was added with normal-RI dNK cells. 40 cells were chosen at random and tracked through the sequence. Cells were scored when obvious apoptotic morphology (transition to a phase bright appearance, decrease in cytoplasmic and nuclear size, and the formation of a membrane bleb or a blister) was initially observed. Area under the curve data (arbitrary units) was generated from each kinetics curve using GraphPad Prism software. The median gestational age of the samples used to generate dNK for VSMC co-culture was 12.2 weeks (range 10.1-13.3) for normal-RI and 10.7 weeks (range 9.3-12.6) for high-RI. The median gestational age of the samples used to generate dNK for EC co-culture was 11.4 weeks (range 9.4-12.7) for normal-RI and 10.4 weeks (range 9.1-11.7) for high-RI.

**References**

**(Note: reference numbers correspond to reference list in main article)**

8. Ashton SV, Whitley GS, Dash PR*, et al.* Uterine spiral artery remodeling involves endothelial apoptosis induced by extravillous trophoblasts through Fas/FasL interactions. *Arterioscler Thromb Vasc Biol* 2005; **25**: 102-108.

9. Harris LK, Keogh RJ, Wareing M*, et al.* Invasive trophoblasts stimulate vascular smooth muscle cell apoptosis by a fas ligand-dependent mechanism. *Am J Pathol* 2006; **169**: 1863-1874.

11. Keogh RJ, Harris LK, Freeman A*, et al.* Fetal-derived trophoblast use the apoptotic cytokine tumor necrosis factor-alpha-related apoptosis-inducing ligand to induce smooth muscle cell death. *Circ Res* 2007; **100**: 834-841.

45. Fickling SA, Tooze JA, Whitley GS. Characterization of human umbilical vein endothelial-cell lines produced by transfection with the early region of Sv40. *Exp Cell Res* 1992; **201**: 517-521.

46. Cartwright JE, Whitley GS, Johnstone AP. The expression and release of adhesion molecules by human endothelial cell lines and their consequent binding of lymphocytes. *Exp Cell Res* 1995; **217**: 329-335.
